# Association between adherence to the dietary approaches to stop hypertension (DASH) diet and maternal and infant sleep disorders

**DOI:** 10.1186/s40795-022-00600-0

**Published:** 2022-09-19

**Authors:** Samira Karbasi, Ehsaneh Azaryan, Alireza Zangooie, Asghar Zarban, Afsane Bahrami

**Affiliations:** 1 Department of Molecular Medicine, School of Medicine, Cardiovascular Diseases Research Center, Birjand University of Medical Sciences, Birjand, Iran; 2 Student Research Committee, Birjand University of Medical Sciences, Birjand, Iran; 3 Cellular and Molecular Research Center, Birjand University of Medical Sciences, Birjand, Iran; 4 Cardiovascular Diseases Research Center, Birjand University of Medical Sciences, Birjand, Iran; 5 Clinical Biochemistry Department, Faculty of Medicine, Birjand University of Medical Sciences, Birjand, Iran; 6 Clinical Research Development Unit of Akbar Hospital, Mashhad University of Medical Sciences, Mashhad, Iran; 7 Clinical Research Development Unit, Imam Reza Hospital, Faculty of Medicine, Mashhad University of Medical Sciences, Mashhad, Iran

**Keywords:** DASH diet, Nutrition, Sleep disorders, Mothers, Infants

## Abstract

**Background:**

Maternal diet is known to be important to both mother and infant health. The purpose of this study was to evaluate the association between adherence to the Dietary Approaches to Stop Hypertension (DASH) dietary pattern (DP) and sleep problems in mothers and their infants.

**Methods:**

The study included 350 breastfeeding mothers with an average age of 29.5 ± 5.9 years. Psychological functions were performed using standard questionnaires, including a Quality-of-Life Questionnaire (QLQ), Edinburgh Postnatal Depression Scale (EPDS), Spielberger Anxiety Questionnaire (SAQ), Pittsburg Sleep Quality Index (PSQI), and Infant Sleep Questionnaire (ISQ). Also, a standardized food frequency questionnaire (FFQ) was used to identify adherence to the DASH DP.

**Results:**

Subjects in the highest tertile of DASH DP had significantly lower scores of mother’s sleep latency (0.70 ± 1.18 vs. 1.24 ± 1.3; *P* value = 0.031), sleep disorders (4.3 ± 1.6 vs. 5.3 ± 2.4; *P* value= 0.032) and higher mother sleep efficiency compared to those in the lowest tertile (97.5 ± 89 vs. 54.8 ± 90; *P* value= 0.011). Also, infants of mothers with higher adherence to a DASH DP had lower sleep disorders compared with subjects with low adherence (4.9 ± 3.8 vs. 5.7 ± 3.2; *P* value= 0.017). After controlling for the mother’s education, economic status, age, body mass index (BMI), and energy intake, adherence to the DASH pattern was associated with shorter sleep latency (β = 0.60; 95%CI: 0.49–0.82), fewer sleep disorders score in mothers (β = 0.92; 95%CI: 0.85–0.99) and their infants (β = 0.90; 95%CI: 0.84–0.96) as well as high mother’s sleep efficiency (β = 1.2; 95%CI: 1.1–1.31).

**Conclusions:**

According to our findings, adherence to DASH DP is associated with a lower score for sleep disorders in mothers and their infants.

## Background

In prenatal and postnatal women, sleep disturbances such as poor sleep are prevalent. For instance, clinically severe insomnia symptoms affect 14–76% of expectant mothers and 87.5% of postpartum women [1]. After childbirth, women’s sleep patterns are disrupted, and they feel more exhausted. In the postnatal period, women are more sleepless than throughout pregnancy and/or previous periods of reproductive age. Sleep disruption in the mother during pregnancy increases the possibility of offspring sleeping problems. Also, maternal short sleep and sleep-disordered breathing during pregnancy raise inflammatory cytokine levels, which can induce infant developmental disorder. There appears to be a bidirectional association between sleep quality and duration with diet at this time [2]. Maternal common mental disorder (CMD), characterized by depression, anxiety, and somatic symptoms, is one of the leading causes of illness burden in low and middle-income societies [3]. Asian research indicates that a mother’s mental illness is a risk factor for poor infant development and growth. Depression and anxiety are frequent among females in pregnancy and the postpartum period, with a recorded incidence of depression of up to 35%. Concertedly called maternal CMD, these conditions are debilitating and frequently chronic [4]. The infants of depressed mothers had lower activity levels and stronger protests, as well as lower body weight. In addition, an interrupted mother’s sleep has been linked to a higher risk of postpartum depression in some studies [5, 6].

The Dietary Approaches to Stop Hypertension (DASH) dietary pattern (DP) was developed in the 1990s and is currently recommended by physicians as one of the therapies for high blood pressure [7]. This diet equates to 6–8 daily servings of grains or grain products, 4–5 daily servings of vegetables and fruit, 2–3 daily servings of low-fat dairy foods, 2 daily servings of meat, poultry, or fish, 2–3 daily servings of fats and oils, 2300 mg/day of sodium, 4–5 weekly servings of nuts, seeds, or dry beans, and 5 weekly servings of sweets for every 2000 cal consumed. DASH DP is a major component of a heart-healthy diet since it reduces salt, saturated fat, and trans-fat intake while increasing magnesium, potassium, calcium, fiber, and protein intake, hence lowering blood pressure [8, 9]. Also, a clinical trial reported that in postmenopausal females, higher adherence to a DASH DP could improve mood and depression symptoms [10]. In 1997, the DASH DP was proposed as a lifestyle modification interference with proven benefit in the management of increased blood pressure. Since then, an increasing body of evidence indicating a connection between hypertension incidence and impaired mental health (e.g., depression, anxiety, and psychosocial problems) has persuaded some researchers to investigate the potential positive effects of this diet on psychological health [11].

On the other hand, understanding how dietary habits affect the sleep quality of mothers and their infants may provide insights into how to further improve the well-being and health of mothers and their babies. Therefore, the current study aimed to investigate the association between adherence to DASH DP and maternal and infant sleep disorders. Secondly, we wished to investigate the association between maternal CMDs and adherence to DASH DP.

## Methods

### Study population

In January–February 2021, 350 mothers and their infants were recruited from 4 different areas of Birjand, South Khorasan, Iran. There were several requirements for participating in this investigation: having an infant aged 1–6 months, not having a history of psychological disorders like depression/anxiety, and age between 20 and 35 years. Mothers who had used anti-inflammatory, antioxidant, and anti-depressant during the previous year were excluded.

### Assessment of emotional function

#### Quality of life (QL) score

The Short Form12-item survey (SF-12) is a brief version of the SF-36 and is one of the common generic tools for the evaluation of the physical and mental aspects of QL. This instrument consists of 12 items covering eight domains of personal health, with higher scores indicating improved health-related QL. This study used the Persian version of the SF-12, which has shown a high level of reliability and validity for Iranians (Cronbach’s alpha for physical and mental health was 0.73 and 0.72, respectively) [12].

#### Depression score

The Edinburgh Postnatal Depression Scale (EPDS) is a 10-item tool that was used to screen probable depression symptoms in the postpartum period. These items were assessed on a 4-point scale to create a score ranging from 0 to 30, with high scores indicating poor mother mood. The reliability and validity of this tool have already been shown in the Iranian population (Cronbach’s alpha for EPDS was 0.79) [13].

#### Anxiety score

The Spielberger Anxiety Questionnaire (SAQ) was used to determine the participant’s anxiety level. This tool consists of two parts that measure the trait and state of anxiety separately. Each section is comprised of 20 questions, every of which has a score ranging from 1 to 4. The total score ranges between 20 and 80 points and is classified into 3 groups: mild (20–40 points), moderate (40–60), and severe anxiety (60–80). The reliability (r = 0.97) and validity of its Persian version were approved in 2007 using a test re-test [14].

#### Maternal sleep disorders score

In this investigation, we utilized the Persian version of the 18-item Pittsburg Sleep Quality Index (PSQI) as validated by Farrahi-Moghaddam et al. [15]. This tool contained 7 subscales: subjective sleep quality, sleep latency, sleep duration, sleep efficiency, sleep disturbance, sleep drug use, and daytime sleepiness, all of which were used to assess the subjective quality of sleep-in individuals during a month. The questions were scored based on a 3-point Likert scale and the sum score of the index was calculated by adding up the scores of the 7 subscales (range: 0–21). Finally, a total score above five shows poor sleep quality. The validity and reliability of this questionnaire have been assessed in Iran (Cronbach’s alpha = 0.83 and correlation coefficient = 0.88) [16].

#### Infant sleep disorders score

The Infant Sleep Questionnaire (ISQ) is a 10-item questionnaire designed to measure infant sleep habits. The length of time it takes to settle an infant to sleep is all answered in three questions. The frequency of night waking is determined by two questions: the number of times each night and the days each week. One question asks about the average time it takes for an infant to get back to sleep, and another item is the duration time of the waking problem. Two questions inquire about the frequency with which parents bring their children into their beds at night. The final question requires parents to identify whether their child has sleeping problems, with responses ranging from “no” to “severe” [17]. The validity and reliability of this questionnaire have been investigated in Iran (Cronbach’s alpha coefficient > 0.70 and correlation coefficient values (r) are greater than 0.5) [18, 19].

#### Evaluation of other variables

A nurse and health care professionals obtained data about the volunteers’ demographic characteristics, economic status, mother age, mother education, maternal body mass index (BMI), infant age, infant sex, and infant head circumference (cm). BMI was calculated by dividing weight (kg) by height (m) squared. With the use of a measuring tape, the infant’s height and head circumference were measured to the nearest millimeter. The weight was also recorded to the closest 0.1 kg using digital scales.

#### Dietary intake assessment

Mothers fulfilled a valid and reliable 65-item semi-quantitative food frequency questionnaire (FFQ) which was asked by trained dieticians. This tool estimates the usual dietary intake for each item in the prior year on a daily, weekly, monthly, monthly, or rarely basis. Diet Plan 6 software was used for food analysis. For Iranians, the validity of this test has been confirmed [20].

#### Adherence to the DASH diet

DASH scores were computed using the approach of Fung et al. [21]. As per quantile categories, the DASH score comprises an indicator of a high intake of nuts and legumes, fruit, vegetables, low-fat dairy foods, and whole grains (i.e., the lowest quantile is considered one point, and the highest quantile is considered five points). Higher points were given to those who consumed the lowest quantile of sugary beverages, sodium, and red and processed meats. Therefore, the total DASH score, which ranges from 8 to 40 points, was calculated by combining all component scores. We performed the analyses and comparisons across tertiles of the total DASH score (range between 8 to 40).

### Statistical analysis

The DASH DP tertiles were used to categorize mothers by using the DASH score. Using the Kolmogorov-Smirnov test, all variables were found to have a normal distribution. The ANOVA and post hoc Tukey test were used to compare continuous variables across tertiles of DASH DP scores, and the results are reported as mean ± standard deviation (SD). The association of tertiles of DASH DP with having any type of psychological disorders in mothers and their infants was assessed using crude and adjusted linear regression models. We accounted for potential confounders including mother’s education, economic status, age, BMI, and energy intake in the adjusted model. A *p*-value < 0.05 was defined as statistically significant.

## Results

The general characteristics of the research subjects are described in Table 1**.** The 350 women with an average age of 29.5 ± 5.9 years were divided into 3 groups based on the tertiles of their DASH DP: T1 was in the lowest tertiles, T2 was in the second, and T3 was in the highest. There’s no significant relationship between the persons’ general anthropometric and demographic information including economic status, mother age, mother education, mother BMI, infant age, infant sex, and infant head circumference for participants in the lowest (T1) and greatest (T3) tertiles of DASH DP adherence (*P* > 0.05).

**Table 1:** Demographic and anthropometric characteristics of study participants

Variables (score)		T1 33.7% (118)	T2 36.1% (126)	T3 30.2% (106)	*P*-value
M Age (y)		29.9 ± 6.2	29.3 ± 6.0	29.2 ± 5.6	0.380^a^
Economic status	Less than enough	26.5%	27%	38.5%	0.528^b^
	Enough	70.5%	71%	60.5%	0.559^b^
	More than enough	3%	2%	1%	0.911^b^
M education (year)	0–9	26.4%	20%	12.9%	0.121^b^
	10–12	42.9%	20%	17.8%	0.423^b^
	> 13	30.7%	60%	69.3%	0.501^b^
M BMI (Kg/m2)		23.8 ± 4.2	25.4 ± 4.2	26.7 ± 3.9	0.213^a^
I Age (day)		127 ± 36.7	135.7 ± 31.4	125 ± 34.2	0.617^a^
I Sex, male, n (%)		128 (55.7%)	134 (53.6%)	80 (46.5%)	0.135^a^
I head circumference (cm)		42.1 ± 2.8	39.4 ± 4.5	39.1 ± 2.6	0.221^a^

Mother (M); Infant (I); Body mass index (BMI)

Data presented as Mean ± SD or number (%)

^a^*p*-value from analysis of the variance (ANOVA) for groups comparison

^b^using chi-square test

As shown in Table 2, subjects who adhered to the DASH style diet consumed more dietary fiber, fruits, vegetables, low-fat dairy products, whole grains, and nuts (*P* < 0.001). The subjects in the 3rd tertile of the DASH diet category (high adherence) consumed significantly less red and processed meat, sugar-sweetened beverages (SSB), and sodium than those in the 1st tertile (bottom adherence; *P* < 0.05).

**Table 2:** Dietary intakes of participants in different tertiles of the adherence to the DASH DP scores

Variables	Tertiles of DASH score			
	T1 33.7% (118)	T2 36.1% (126)	T3 30.2% (106)	*P*-value ^a^
**Dietary nutrient intake**				
Total energy (kcal)	2180 ± 640	2156 ± 758	2077 ± 881	0.311
Carbohydrate (g/1000Kcal)	200.81 ± 79.52	197.57 ± 74.11	258.66 ± 98.20	0.143
Protein (g/1000Kcal)	61.31 ± 14.46	56.95 ± 14.93	73.90 ± 14.11	0.310
Fat (g/1000Kcal)	131.91 ± 40.80	132.57 ± 41.22	160.35 ± 59.21	0.437
Dietary fiber (g/1000Kcal)	20.51 ± 3.25	20.81 ± 3.11	26.84 ± 3.72	**0.011**
Zinc (mg/1000Kcal)	7.59 ± 1.39	7.23 ± 1.20	9.51 ± 1.10	0.302
Folate (μg/1000Kcal)	24.31 ± 35.22	25.71 ± 32.76	34.48 ± 40.71	0.137
**Components of DASH DP (serving/1000 Kcal)**				
Fruits (serving/1000 Kcal)	0.24 ± 0.43	0.48 ± 0.50	0.78 ± 0.33	**< 0.001**
Vegetables (serving/1000 Kcal)	0.51 ± 0.85	0.64 ± 0.92	0.84 ± 0.97	**< 0.001**
Nuts, legume, seed (serving/1000 Kcal)	2.10 ± 1.22	3.08 ± 1.16	4.23 ± 1.11	**< 0.001**
Low fat dairy (serving/1000 Kcal)	0.14 ± 0.22	0.15 ± 0.27	0.39 ± 0.74	**< 0.001**
Whole grain (serving/1000 Kcal)	2.51 ± 2.0	4.02 ± 2.81	4.55 ± 2.47	**< 0.001**
Red and processed meat (serving/1000 Kcal)	0.51 ± 0.48	0.42 ± 0.46	0.41 ± 0.53	**0.041**
Sweetened beverage (serving/1000 Kcal)	0.18 ± 0.70	0.17 ± 0.36	0.10 ± 0.26	**< 0.001**
Sodium (mg/1000Kcal)	1566 ± 540	1421 ± 812	1376 ± 609	**0.050**

Data presented as Mean ± SD

^a^Obtained from ANOVA test

The relationships between DASH diet style compliance and neuropsychological parameters are presented in Table 3. Adherence to the DASH pattern was associated with a shorter sleep disorder score in mothers and their infants, as well as a high mother’s sleep efficiency (*P* < 0.05).

**Table 3:** Neuropsychological parameters of study participants by tertiles (T) categories of DASH DP score

Variables (score)	Tertiles of DASH score			
	T1 33.7% (118)	T2 36.1% (126)	T3 30.2% (106)	*P* value ^a^
**Edinburgh post-natal depression**				
Edinburgh depression	7.1 ± 3.4	7.4 ± 3.2	7.4 ± 2.9	0.831
**Quality of life**				
Physical health	15.1 ± 2.5	15 ± 2.7	15.3 ± 2.5	0.746
Mental health	0.75 ± 0.68	0.83 ± 0.53	0.84 ± 0.54	0.442
**Spielberger State – Trait Anxiety Inventory**				
Anxiety	29.2 ± 11.1	30.1 ± 9.6	31.8 ± 11.1	0.350
**Mother sleep**				
Sleep latency	1.24 ± 1.3	0.86 ± 1.2	0.70 ± 1.18	0.031***
Sleep efficiency	54.8 ± 90	69.5 ± 110	97.5 ± 89	0.011**
Need for sleep medications	0.059 ± 0.30	0.031 ± 0.21	0.023 ± 0.15	0.491
Daytime dysfunction	0.25 ± 0.92	0.17 ± 0.52	0.30 ± 0.78	0.457
Sleep disorders total score	5.3 ± 2.4	5.1 ± 1.6	4.3 ± 1.6	0.032**
**Infant sleep**				
Infant sleep disorders total score	5.7 ± 3.2	4.4 ± 3.3	4.9 ± 3.8	0.017**

^a^p-value from analysis of the variance (ANOVA) for groups comparison

^*^1–2* p *< 0.05

^**^1–3* p* < 0.05

^***^2–3 *p* < 0.05

Results from linear regression analysis for neuropsychological disorders across tertiles of DASH diet scores are presented in Table 4. After controlling for the mother’s education, economic status, age, BMI, and energy intake, participants with the highest tertile DASH score were less likely to have sleep latency (β = 0.60; 95%CI: 0.49–0.82; *p* < 0.001,) lesser sleep disorders score in mothers (β = 0.92; 95%CI: 0.85–0.99; *p* < 0.05) and their infants (β = 0.90; 95%CI: 0.84–0.96; *p <* 0.05) as well as high mother’s sleep efficiency (β = 1.2; 95%CI: 1.1–1.31; *p* < 0.05) compared to the lowest tertile. Also, no significant difference was observed between the highest tertile compared to lowest tertile of DASH score regarding to the Edinburgh depression (β = 0.62; 95%CI: 0.30–1.28; *p* > 0.05), physical health (β = 1.9; 95%CI: 0.78–3.42; *p >*  0.05), mental health (β = 1.84; 95%CI: 0.74–3.21 *p >* 0.05), anxiety (β = 0.70; 95%CI: 0.34–1.47; *p>* 0.05), need for sleep medications (β = 1.32; 95%CI: 0.56–3.02; *p >*0.05) and daytime dysfunction (β = 0.89; 95%CI: 0.43–1.84 *p >* 0.05).

**Table 4:** Linear regression analysis for neuropsychological disorders across tertiles of DASH DP scores

Variables	Tertiles of DASH score β (95% CI)		
	T1 33.7% (118)	T2 36.1% (126)	T3 30.2% (106)
Edinburgh depression	Ref.	1.07(0.52–2.22)	0.62(0.30–1.28)
Physical health	Ref.	0.90(0.44–1.86)	1.9(0.78–3.42)
Mental health	Ref.	0.63(0.26–1.12)	1.84(0.74–3.21)
Anxiety	Ref.	0.32(0.14–0.71)	0.70(0.34–1.47)
Sleep latency	Ref.	0.70(0.58–0.85) **	0.60(0.49–0.82) ***
Sleep efficiency	Ref.	1.1(0.99–1.5)	1.2(1.1–1.31) *
Need for sleep medications	Ref.	1.42(0.42–2.44)	1.32(0.56–3.02)
Daytime dysfunction	Ref.	0.91(0.39–1.67)	0.89(0.43–1.84)
Mother sleep disorders total score	Ref.	0.72(0.37–1.13)	0.92(0.85–0.99) *
Infant sleep disorders total score	Ref.	0.70(0.34–1.47)	0.90(0.84–0.96) *

Tertile 1 was considered as the reference group

Adjusted for mother’s education, economic status, age, BMI, and energy intake

*p < 0.05

**p < 0.01

***p < 0.001

## Discussion

We found that high adherence to DASH DP is associated with a lower risk of sleep disturbance in mothers and their infants. However, we found no correlations between DASH DP and depression, QL, and anxiety scores in mothers. To the best of our knowledge, this is the first study that has comprehensively assessed the correlation between adherence to the DASH diet and psychological function in mothers and their infants.

In the first 6 months after giving birth, women are highly stressed and sensitive as they focus on integrating a new person into the family. Sleep deprivation, sleeplessness, and exhaustion are usually the concerns that women face during this period [5]. Sleep disruptions (altered mothers’ sleep patterns), particularly in the early months, are linked to overnight infant feeding, nursing, and sleeping habits. The DP and lifestyle changes were recommended as one method of preventing sleep problems [22]. In two cross-sectional investigations performed on moms with infants, low sleep duration or quality was correlated with poorer diet quality [23, 24]. According to Pahlavani and colleagues, the DASH diet is important for decreasing insomnia in individuals [25]. Also, another report revealed that consuming a low-inflammatory-index diet like the DASH DP increased the quality of sleep [26]. Previous studies indicated that sleep disruption could cause an accumulation of free radicals in the cerebral tissues, and increase cerebral oxidative stress [27]. DASH DP can lower oxidative stress, which may help support the significance of our findings and the potential effectiveness of the DASH DP in preventing insomnia [28].

A study of Japanese employees found that consuming a healthy diet rich in fruits, vegetables, whole grains, chicken, fish, soya drinks, and eggs, as well as reduced-fat dairy products, was linked to a lower incidence of sleep problems. The findings of Chen Bai et al. revealed that vegetable eating could be a preventive factor in reducing the negative effects of irregular sleep [29]. Low fiber/carotenoid consumption has been related to poor sleep quality and an increased risk of insomnia. Cross-sectional research among people in the United Kingdom found a correlation between fruit/vegetable intake and sleep wellness, with deep sleepers having higher vitamin C plasma levels [30]. Additionally, another study compared those who slept for short periods to those who slept for prolonged periods and found that short-sleepers absorbed fewer vitamins C and D. A variety of vitamins, such as vitamin D, are associated with changes in sleep quality through altered disorders such as restless legs syndrome and obstructive sleep apnea syndrome. Vitamin D is found naturally in fish. Vitamin D is also involved in the creation of melatonin, a hormone that regulates human circadian rhythms and sleeps patterns [31]. Hansen et al. reported that eating fish had a positive influence on sleep and daily functioning, which they linked to vitamin D deficiency [32]. There are positive significant associations between nocturnal sleep disturbance and dietary fat consumption in pregnant American women, as well as shorter time to fall asleep and higher fruit and vegetable intake. Increased saturated fat intake during the day was associated with a shorter duration of slow-wave sleep and more arousals during the night in a survey of normal-weighted adults [33]. Another investigation on 459 postmenopausal women looked into the relationship between dietary components and sleep. The authors concluded that total sleep time was negatively related to whole and saturated fat intake. Low fiber consumption and high saturated fat and sugar intake are also related to lighter, less sleep, and increased alertness [33].

A prospective investigation of a considerably larger group of postmenopausal women found that a high glycemic index (GI) and glycemic load diets are related to an increased prevalence of insomnia, as was high consumption of dietary added sugars and starch. They also showed that hyperglycemia caused by a high-GI diet and compensatory hyperinsulinemia could trigger the production of autonomic counter-regulatory hormones such as adrenaline, cortisol, glucagon, and growth hormone, all of which could lead to insomnia [34].

Milk and dairy products are typically thought to improve sleep quality and have a positive impact on both physical and mental health. A well-balanced diet including milk and dairy products helps enhance sleep quality. The mechanism of action is thought to be that a high concentration of tryptophan, which is required to generate melatonin, found in milk and dairy products can inhibit the inhibitory neurotransmitter gamma-aminobutyric acid [35].

Tanaka and colleagues suggested that low protein consumption (< 16% of energy from protein) was correlated to poor sleep quality and difficulties initiating sleep, whereas high protein intake (> 19% of energy from protein) was associated with high sleep quality [33].

Recently, the need for increasing knowledge of the correlation between high sleep quality and healthy DP in mothers has been highlighted. Improved sleep quality may promote mother and infant health [36]. Some nutrients, including tryptophan, nucleotides, essential fatty acids, and omega-3 long-chain fatty acids, have been shown to naturally fluctuate in a mother’s breast milk with circadian rhythm, and these nutrients are vital for influencing infant sleep. Furthermore, sleep duration has been connected to micronutrient sufficiency, with Fe, Zn, and Mg levels in infants being positively associated with sleep duration [37].

To the best of our knowledge, it is the first study that has investigated the association between adherence to the DASH DP and maternal and infant sleep disorders. It should also be noted that our study’s strengths include an appropriate sample size of participants and a comprehensive examination of confounding variables. Nonetheless, there are certain limitations to our study that should be noted. Firstly, because this is a cross-sectional study, the temporality of associations cannot be validated. Secondly, self-reporting questionnaires used to measure sleep disorders are likely to be susceptible to misreporting.

## Conclusion

We found that greater adherence to the DASH DP is associated with a reduced risk of sleep disorders in both mothers and their infants. More research, particularly longitudinal studies, is needed to confirm these findings in a larger population and to determine whether intervention through dietary change is a practical solution.

## Data Availability

The datasets used and/or analyzed during the current study are available from the corresponding author on reasonable request.

## Abbreviations

DASH: Dietary Approaches to Stop Hypertension
DP: Dietary pattern
QL: Quality of life
FFQ: Food frequency questionnaire
CMD: Common mental disorder
SF-12: Short Form12-item survey
EPDS: Edinburgh Postnatal Depression Scale
SAQ: Spielberger Anxiety Questionnaire
PSQI: Pittsburg Sleep Quality Index
ISQ: Infant sleep questionnaire
SD: Standard deviation
BMI: Body mass index
SSB: Sugar-sweetened beverages
GI: Glycemic index
